# Patient-Centered Discussion on End-of-Life Care for Patients with Advanced COPD

**DOI:** 10.3390/medicina58020254

**Published:** 2022-02-08

**Authors:** Holly Mitzel, Dakota Brown, Morgan Thomas, Byrne Curl, Mackenzie Wild, Andrea Kelsch, Judge Muskrat, Abulquasem Hossain, Ken Ryan, Olawale Babalola, Madison Burgard, Masfique Mehedi

**Affiliations:** School of Medicine and Health Sciences, University of North Dakota, Grand Forks, ND 58202, USA; holly.j.mitzel@und.edu (H.M.); dakota.s.brown@und.edu (D.B.); morgan.thomas@und.edu (M.T.); byrne.curl@und.edu (B.C.); mackenzie.wild@und.edu (M.W.); andrea.v.kelsch@und.edu (A.K.); judge.muskrat@und.edu (J.M.); abulquasem.hossain.1@und.edu (A.H.); ken.ryan@und.edu (K.R.); olawale.babalola@und.edu (O.B.); madison.r.burgard@und.edu (M.B.)

**Keywords:** chronic bronchitis, chronic obstructive pulmonary disease, spirometry, exacerbation, bronchodilators, end-of-life care, palliative care, global initiative for chronic obstructive lung disease, β_2_-agonists, corticosteroids, dyspnea, emphysema

## Abstract

Exacerbations of chronic obstructive pulmonary disease (COPD) may lead to a rapid decline in health and subsequent death, an unfortunate tyranny of having COPD—an irreversible health condition of 16 million individuals in the USA totaling 60 million in the world. While COPD is the third largest leading cause of death, causing 3.23 million deaths worldwide in 2019 (according to the WHO), most patients with COPD do not receive adequate treatment at the end stages of life. Although death is inevitable, the trajectory towards end-of-life is less predictable in severe COPD. Thus, clinician-patient discussion for end-of-life and palliative care could bring a meaningful life-prospective to patients with advanced COPD. Here, we summarized the current understanding and treatment of COPD. This review also highlights the importance of patient-centered discussion and summarizes current status of managing patients with advanced COPD.

## 1. Introduction

Chronic obstructive pulmonary disease (COPD) affects more than 10 million people in the United States [1,2]. COPD is a diseased state in which an individual experiences irreversible airflow limitation and persistent respiratory symptoms. COPD pathophysiology includes emphysema, chronic bronchitis, and other minor airway disorders. COPD is more prevalent among smokers than non-smokers and is more prevalent among men than women [1]. Though men have a higher prevalence than women overall, women are more likely to require hospitalization for severe disease cases due to anatomical and physiological differences [3]. Importantly, the presence of comorbidities impacts COPD patients negatively, increasing the likelihood of hospitalization and mortality and reducing the quality of life [4,5,6].

The current general clinical management of COPD is aimed at treating and preventing acute exacerbation, focusing on the underlying pathophysiology, e.g., treating bronchoconstriction, reducing hyperinflation, and airway inflammation [7]. Unfortunately, advanced COPD is often a progressive and terminal illness [8]. The optimal management of symptoms in patients with advanced COPD is often neglected under the current COPD disease management model. However, the total traditional therapy for advanced COPD produces a modest relief of symptoms, leaving these patients vulnerable and substantially reducing the health-related quality of life [9,10,11]. When needed the most, many patients with advanced COPD receive inadequate end-of-life care, probably due to the lack of patient-physician communication and uncertainty in predicting prognosis for patients with COPD [8]. The purpose of this review is to summarize pathophysiology, pathogen-induced disease exacerbation, pharmacological management, and recent research regarding end-of-life care for patients with advanced COPD.

## 2. Diagnosis and Classification of COPD

As physical examination findings may not necessarily be sensitive enough for initial COPD diagnosis, clinicians should include a detailed medical history for COPD diagnosis. The global initiative for chronic obstructive lung diseases (GOLD) report is a good source for this information. Additionally, the impacts of disease on patients including limitation of activity, missed work and economic impact, effect on family routines, feeling of depression or anxiety, well-being, and sexual activity should be considered [1]. Overall, the diagnosis of COPD should emphasize three significant aspects: symptoms (e.g., shortness of breath, chronic cough, and sputum), risk factors (e.g., host factors, tobacco, occupation, indoor or outdoor pollution), and spirometry (pre- and post-bronchodilator pulmonary function tests) [1]. A positive diagnosis for COPD patients is generally attributed to dyspnea, chronic cough (can be productive or unproductive) or mucus production, a history of recurrent lower respiratory infections, and a history of exposure to COPD risk factors [1]. Importantly, spirometry is required to make the diagnosis. Spirometry measures the FEV1 (forced expiratory volume within one second) and FVC (forced vital capacity). In individuals with asthma, the post-bronchodilator test will indicate an increase in FEV1 by 200 mL and 12% of the initial value. Thus, the airway obstruction is deemed reversible. In contrast, in individuals with COPD, the post-bronchodilator test will not improve FEV1, values remain markedly decreased, and airway obstruction is considered irreversible [12]. The presence of a postbronchodilator FEV_1_/FVC < 0.70 confirms the presence of persistent airflow limitation or COPD [1]. Additional investigative tests include imaging studies such as X-ray or computed tomography, oximetry and arterial blood gas measurements, lung volumes and diffusing capacity, and exercise testing and assessment of physical activity [1]. Although imaging is not diagnostic, it helps exclude other differential diagnoses, such as pneumonia. In addition, imaging studies can reveal pathological changes typically seen in individuals with obstructive lung disease, such as hyperinflated lungs (i.e., barrel chest) or a flattened diaphragm. Thus, proper diagnosis is essential in determining if a patient has COPD. In addition, it is crucial for defining the severity of the disease.

COPD ranges in severity from mild (stage I) to very severe (stage IV) and affected patients can experience unpredictable exacerbations of their symptoms. The Global Initiative for Chronic Obstructive Lung Disease (GOLD) is the standard reference for stage determination based on spirometry (pulmonary function test) results. All stages consist of an FEV1/FVC ratio of <0.70. The primary determinant of a patient’s GOLD stage of COPD severity is an individual’s FEV1 predicted value [2]. Clinicians should also consider physical exam findings during COPD staging. Mild cases may present with no abnormalities on the physical exam, while very severe cases may offer a broader range of symptoms. Individuals with severe to very severe COPD typically present with a chronic cough, pursed-lip breathing, decreased breath sounds, and wheezing or crackles [2]. Weight loss or weight gain may also be noted depending on the severity of COPD and the underlying chronic disease. One important COPD symptom is dyspnea, which is breathlessness on exertion and reduced exercise capacity resulting from the reduced inspiratory capacity. Moreover, reduced airflow on exhalation creates air trapping, which leads to reduced inspiratory capacity [13,14]. Patients with advanced COPD may show alveolar hypoxia, which may turn into hypoxemia (low blood oxygen levels) [14,15]. Additionally, COPD symptoms, include hypercapnia (raised blood carbon dioxide levels): results from reduced airflow-induced impaired gas transfer by damaged alveolar structure and pulmonary vascular bed [14].

## 3. Pathophysiology of COPD

COPD results from the concurrence of peripheral airway inflammation and narrowing of the airways, leading to substantial airflow obstruction, the destruction and loss of alveoli, terminal bronchioles, and surrounding capillary vessels and tissues. The block of airflow may lead to irreversible lung pathophysiology, e.g., emphysema: the destruction of walls between air sacs resulted in larger but fewer air sacs [14]. Chronic inflammation contributes to the hyperplasia of goblet cells, hypertrophy of mucous glands, and a simultaneous decrease in serous cell activity, resulting in increased mucus viscosity. Other changes in respiratory epithelium include a reduction in mucin glycosylation, decreased antimicrobial activity, and increased acidity. The underlying pathologic processes affecting lung parenchyma result in chronic obstructive bronchiolitis with fibrosis, obstruction of small airways, and emphysema with enlargement of airspaces and mucus hypersecretion [16]. Progressive breakdown of alveolar ECM by macrophages and T cells results in the characteristics changes seen in the late stages of COPD. Lung epithelium also demonstrates increased apoptosis due to activation of senescent fibroblasts, which no longer support matrix remodeling [17].

## 4. Pathogen-Induced COPD Exacerbations

In COPD patients, the most common cause of an exacerbation is an infection in the lungs or airways (source, e.g., breathing tubes). Importantly, the exacerbation can also occur from inhaling severe allergen or irritating substances from the environment such as air pollution. Infection (virus, bacterium, or fungus) or allergen exposure to the lung creates inflammation that makes the airways narrow from muscle tightness, swelling, and mucus [18]. The hallmark of COPD is chronic airway inflammation, which causes lung damage and weakens the immune system. This immune dysfunction can lead to COPD exacerbations from various sources, but respiratory pathogens are the most prominent [19]. Acute exacerbations lead to prolonged hospitalization and increased mortality rates of patients with COPD [20]. Approximately 75% of COPD patients have at least one episode of exacerbation per year and episodes are linked to adverse health effects as well as increased morbidity and mortality [21]. Most exacerbations (50–70%) are due to respiratory infections via bacteria, viruses, and atypical organisms [22]. About 10% of exacerbations are caused by environmental pollutants such as particulate matter (≤10 μm diameter), ozone, and nitrogen dioxide [23]. The remaining 30% of exacerbations are of unknown etiology [24]. Co-infection with bacteria and viruses is common [25].

Nontypeable *Haemophilus influenzae*, *Haemophilus parainfluenzae*, *Streptococcus pneumoniae*, *Moraxella catarrhalis*, and *Pseudomonas aeruginosa* are the commonly isolated bacteria in sputum culture studies on COPD exacerbations [25,26]. Approximately 4–5% of exacerbations result from atypical bacteria, such as *Mycoplasma pneumoniae* and *Chlamydophila pneumoniae*. Bacterial infections are associated with more significant episodes of inflammation and risk of exacerbation [25]. Bacteria cause exacerbations through various mechanisms such as inducing mucus hypersecretion, ciliary damage, damage of tissue during adherence, inducing leukocyte recruitment, oxidative stress, and induction of excess protease secretions. Some bacteria incite more robust immune responses than others, but the bacterial load is proportional to inflammation regardless of the bacterial pathogen [27].

The worldwide prevalence of viral infections in acute exacerbated COPD is about 40%. The primary viral pathogens identified include rhinovirus, influenza A, and respiratory syncytial virus (RSV). Metapneumovirus, coronavirus, adenovirus, and parainfluenza viruses are also known to contribute to COPD exacerbation [28,29]. Respiratory viruses are known to utilize the host’s cellular mechanisms for their proliferation and survival. These mechanisms include apoptosis, autophagy, necroptosis, and pyroptosis [30,31]. The mechanisms utilized depend on the causative virus. Influenza viruses are apoptosis inducers early on in infection, while pyroptosis activity becomes more prominent in late-stage influenza infection [32]. RSV decreases the levels of p53 to inhibit apoptosis in affected cells, prolonging the survival of the epithelial cells of the airway [33]. RSV also induces autophagy by producing ROS and activating the AMP-activated protein kinase/mammalian target of rapamycin (AMPK-mTOR) signaling pathway in epithelial cells [34]. Viruses may also damage epithelial cells by changing the cell’s barrier function against external agents or directly killing the epithelial cells, which can aid in bacterial colonization. COPD exacerbations caused by bacterial infections appear to be less severe than COPD exacerbations caused by viral and viral–bacterial coinfections [35,36]. This seems to be evident in rhinovirus coinfections due to the production of proinflammatory cytokines and more severe symptoms with prolonged recovery times [37,38].

A drug dose study for critically ill patients with acute exacerbation of COPD found that the frequency of fungal infections is 4.4% [39]. Another study investigating acute exacerbations of COPD, a fungal population primarily made up of *Candida*, *Phialosimplex*, *Aspergillus*, *Penicillium*, *Cladosporium*, and *Eutypella* was reported [40]. *Aspergillus* has been associated with COPD infections with decreased lung function [41]. Patients with underlying conditions such as COPD are more susceptible to fungal infections due to (a) frequent hospitalization and antibiotic treatment, favoring the growth of fungal pathogens, (b) the common use of long-term or repeated short-term steroids resulting in additional immunosuppression, (c) structural changes of the lung due to pulmonary disease, and (d) comorbidities [42].

The ongoing severe acute respiratory syndrome coronavirus 2 (SARS-CoV-2) outbreaks have taken a toll on patients with COPD; in fact, COPD is one of the high-risk factors for severe illness associated with COVID-19 [43,44,45]. A recent preprint study suggests that SARS-CoV-2 preferentially infects mucus-producing cells, e.g., goblet cells. SARS-CoV-2 replicates better in COPD airway epithelium compared to that of healthy donors probably due to the goblet cell hyperplasia associated with COPD. They also suggested that SARS-CoV-2 infection induced disease exacerbation (e.g., syncytia formation and cell sloughing) in the COPD airway epithelium. Additionally, SARS-CoV-2 infection increases the height of the airway epithelium due to an increment of squamous metaplasia [46,47]. It has been reported that squamous metaplasia is one of the histopathological findings in SARS-CoV-2-infected patient fatalities [48].

## 5. Treatments of COPD

The goal of treatment is to improve symptoms, quality of life, and decrease exacerbation and decrease mortality. For patients with advanced COPD, one aspect of the treatment is to increase the rate of inflow and outflow through the airways and to reduce exacerbations of the disease [49,50]. There are multiple drugs that have proven effective in the management of mild to severe COPD.

β_2_ agonists are viable and effective bronchodilation choices for the treatment of COPD as they can help relieve patients of exacerbation symptoms as well as prove effective in the prevention of exacerbations [49,50,51]. These agonists bind to β_2_-adrenergic receptors on smooth muscle cells in the airway and increase adenylyl cyclase which stimulates the relaxation of the smooth muscle [52]. This smooth muscle relaxation opens the airways and improves airflow. β_2_ agonists are generally split into two main groups: short-acting (albuterol, levalbuterol) and long-acting (formoterol, salmeterol). The short-acting β_2_ agonists (SABA) are mainly used for first-line treatment of acute attacks and exacerbations while the long-acting β_2_ agonists (LABA) are primarily used in more severe cases where the patient will benefit from prophylactic bronchodilation, such as when patients have severely impaired lung function (FEV_1_ < 60%). The LABA are often paired with long-acting muscarinic antagonists (LAMA) for even greater prevention of exacerbations [50]. The general use of β_2_ agonists changes with the course of the disease. In more stable/mild cases, a short-acting β_2_ agonist used when needed is generally adequate. As the disease progresses, a SABA and multiple LABA or anticholinergic medications are used for both prevention and management of exacerbations [49,52]. The use of β_2_ agonists does not come without its own considerations, especially in longer-acting prophylactic use. Because β_2_ agonists also affect the cardiovascular system, they should be used cautiously in patients who also have heart conditions [50,53]. It has been shown to increase sinus tachycardia as well as precipitate more severe heart issues such as arrhythmias, ischemia, and congestive heart failure [53]. These negative considerations are important to understand when making decisions for prophylactic β_2_ agonists.

Muscarinic antagonists, or anticholinergics, serve as bronchodilators that can be implemented independently or in combination with β_2_ agonists to improve symptoms of COPD and reduce the frequency of exacerbations in more severe cases [54]. These agents competitively bind to postganglionic muscarinic receptors within the bronchial tree, inhibiting the activity of acetylcholine and decreasing the levels of cyclic guanosine monophosphate. This limits the parasympathetic constriction of the lung airways and allows for greater airflow [54,55]. Muscarinic antagonists used for COPD can also be organized into short-acting (Ipratropium, Oxitropium) and long-acting agents (Tiotropium). Short-acting muscarinic agents (SAMA) are considered as effective or nearly as effective as β_2_ agonists, and both can be utilized separately for acute symptoms or together to provide relief in severe circumstances. Long-acting antimuscarinic agents are used to treat persistent dyspnea, improve functional capacity, and prevent exacerbations, sometimes independently or with long-acting β_2_ agonists. Due to similar levels of efficacy, antimuscarinic agents are commonly used as alternative treatments for patients who are resistant to sympathomimetic drugs [55,56]. Certain considerations are warranted with the usage of antimuscarinic agents for treating COPD. Because inhaled antimuscarinic agents have little systemic absorption, they are much preferred over oral options to avoid collateral damage. Common side effects reported consist of dry mouth, headache, and nausea, with rare reports of urinary retention, tachycardia, and blurred vision with long-term usage. Due to these potential outcomes, caution is recommended for patients with a risk of glaucoma, benign prostatic hyperplasia, and adverse heart conditions [56,57].

The utilization of corticosteroids (fluticasone propionate) in COPD patients may not have a consensus in the medical field, yet still represents an important option for treatment under specific circumstances [56]. Corticosteroids primarily bind to intracellular receptors that promote the activity of histone deacetylase, thus leading to chromatin compaction and preventing histone acetyltransferase activity. This action silences multiple inflammatory genes, reducing the inflammatory response expressed in many COPD cases. Corticosteroids are also believed to prevent down-regulation of β_2_ adrenoreceptors in response to excessive β_2_ agonists; in turn, β_2_ agonists have been indicated to promote glucocorticoid receptor translocation to the nucleus. This synergistic activity provides a significant clinical improvement in patients compared to monotherapeutic use of corticosteroids [58]. Despite the airway inflammation present in COPD, there is only a modest response seen through corticosteroid monotherapy with suggestion towards the reduction in acute exacerbations [56]. In addition to local adverse effects such as oral thrush and dysphonia, as well as systemic adverse effects with extensive use such as severe osteoporosis and pneumonia, monotherapy usage is generally limited to inhaled form and specific to patients with two or more exacerbations per year [58]. Withdrawal studies also indicate that there is an increase in the rate of exacerbations after cessation of inhaled corticosteroid regimen compared to patients who have not taken corticosteroids, which physicians must consider during long-term treatment [58,59].

The synergistic interaction between corticosteroids and β_2_ agonists has led to a combination therapy that is widely prescribed for COPD [54,60]. Inhaled corticosteroids and long-acting β_2_ agonists have been shown to have improved bronchodilator activity and a reduced annual COPD exacerbation rate. Noting the adverse effects of corticosteroids, clinicians should weigh the benefits of the combined therapy with the potential risks before prescribing treatment [60]. Methylxanthines (Theophylline) serve as pharmacological agents for the treatment of COPD due to their bronchodilator properties [56,61]. Through non-competitive inhibition of phosphodiesterase (increasing cyclic adenosine monophosphate and cyclic guanosine monophosphate, signaling bronchial smooth muscle relaxation) and histone deacetylase activation (inhibition of inflammatory genes), methylxanthines are believed to improve airflow. However, other bronchodilators are preferred due to toxicities at high doses resulting in complications such as life-threatening arrhythmias, cardiac arrest, or seizures. Administration involves extensive monitoring and titrating up to therapeutic doses at the lowest possible rate [61]. There is success in using triple therapy in the management of COPD. The clinical evidence suggests that triple therapy is the most effective treatment in reducing the risk of exacerbation in patients with advanced COPD [62]. Triple therapy consists of LAMA, LABA, and inhaled corticosteroid (ICS). Triple therapy can be in multiple inhalers “open triple”. For example, adding a LAMA to a fixed ICS/LABA or vice versa. Triple therapy can be in one inhaler. For example, two different combinations of ICS/LABA/LAMA in a single inhaler. The first combination includes beclomethasone dipropionate/formoterolfumurate/glycopyrronium bromide (BDP/FF/G; TRIMBOW^®^, Chiesi Farmaceutici SpA, Parma, Italy) and the second combination includes fluticasone furoate/vilanterol/umeclidinium (FLF/VI/UMEC; TRELEGY ELLIPTA^®^, GlaxoSmithKlaine, Brentford, UK). BDP/FF/G has been developed as an extra fine formulation, which is composed of aerosol particles with a mass median aerodynamic diameter (<2 µm). It provides 87/5/9 µg of BDP/FF/G when delivered by a pressurized metered-dose inhaler (two inhalations twice daily). In contrast, FLF/VI/UMEC has been developed as a multidose dry-powder inhaler (MDDPI) formulation. It provides 92/22/55 µg of FLF/VI/UMEC when delivered by the ELLIPTA device in each single inhalation (one inhalation daily) [62]. The use of triple therapy showed threefold benefits (a lower rate of moderate and severe exacerbation of COPD, better lung function, and better health-related quality) compared to double therapy or monotherapy in patients with advanced COPD [63]. There is a third triple therapy at the advanced development stage and recent clinical data on it are encouraging. The third combination includes budesonide/glycopyrronium/formoterol (B/G/F). The BGF triple therapy improved symptoms and health-related quality of life in patients with moderate to severe COPD [64,65].

Mechanical ventilation, which increases pulmonary gas exchange, has been using COPD treatment. It is particularly helpful for recovering from the fatigue state of the compromised respiratory muscle [66]. Non-invasive positive-pressure ventilation (NPPV) is generally the first line of treatment for COPD patients. However, invasive positive-pressure ventilation may be required for patients with advanced COPD [66]. A portable noninvasive open ventilation system (NIOV) assists breath-actuated, total volume augmentation and oxygen to adults with imbalance respiratory insufficiency. This portable NIOV system offers a practical option for increasing physical activity levels in COPD patients [67]. Home ventilators are different from both continuous positive airway pressure (CPAP) and bilevel positive airway pressure (BiPAP) ventilators. Importantly, home ventilators keep patients healthy and away from hospitalization [68]. It was previously known that long-term oxygen supply can prolong the life of patients with COPD [69]. Indeed, a recent study suggests long-term home oxygen therapy can improve survival of patients with advanced COPD, particularly the patients with severe hypoxemia (arterial PaO_2_ less than 55 mm Hg (8.0 kPa) [70].

Many patients with COPD die due to their comorbidities rather than COPD. Indeed, the risk of mortality can be increased by two-fold due to more than one comorbidity [6]. A wide range of diseases are known to contribute to the burden of severe disease and mortality in patients with COPD. Iglesias et al. described an expert recommendation for the management of COPD patients with comorbidities. Moreover, Franssen et al. described personalized medicine for patients with COPD [5,71].

The most effective preventative measures that can be taken to protect an individual from developing COPD are avoidance of tobacco smoke and limiting exposure to environmental irritants [72]. Other preventative measures, such as vaccinations against respiratory infections, physical activity, and nutrition are effective in preventing disease [72]. Because the disease process occurs over many years and is difficult to reverse, primary prevention is the most effective way to reduce and prevent the burden of COPD [72].

## 6. Patients Centered Conversation on End-of-Life Care

The debilitating symptoms of COPD have a significant impact on the lives of patients with COPD and lead to decreased quality of life [73]. These symptoms include breathlessness, cough, pain, anorexia, fatigue, and psychological symptoms [74]. The burden of these symptoms is high and thus patients with advanced COPD symptoms should be informed of the benefits of end-of-life and palliative care. Palliative care not only reduces COPD symptoms leading to increased quality of life, but can also decrease the use of invasive procedures, increase psychosocial support and mood, and decrease health care costs. Many patients may also benefit from the early introduction of palliative care before severe symptoms are present [73]. As each person’s COPD is different, a patient-centered management plan is important. A patient-centered COPD treatment team may include a primary care physician, pulmonologist, respiratory therapist, dietician or nutritionist, therapist or counselor, and palliative care specialist [75,76]. Patients’ family members need to be involved as part of the treatment team for COPD patients, as they can be an extension of the physician’s voice. Family members and caregivers can be very instrumental in monitoring COPD patients’ treatment management plan by making sure the patient takes his or her medications, recognizes signs of exacerbation, and helps them to carry out tasks in the home environment. For patients with advanced COPD, a family member can act on behalf of the patient [77].

However, the lack of an effective end-of-life care strategy for patients with advanced COPD may bring dissatisfaction to the patients and relevant stakeholders associated with the treatment and palliative care. One of the main reasons for dissatisfaction or poor outcome of end-of-life care is the lack of good communication between patients, families, and physicians while planning for the advanced directives [8,78]. Studies show that advanced directives are useful among patients with COPD because of their likely illness, potential risk for severe illness, and sudden death of critically ill patients due to disease exacerbation [79]. In some cases, patients with COPD may have an opinion about the decision to forgo CPR and mechanical ventilation for acute respiratory failure [80]. Several studies have suggested that advanced directives can reduce stress among family members of dying patients [81,82].

Many clinicians acknowledge the importance of end-of-life planning early in the process of COPD, however; for chronic respiratory diseases, such as COPD, advanced care planning is rare [83]. Furthermore, it appears that patients diagnosed with COPD often “underutilize” palliative care when compared to other lung diseases such as lung cancer and thus may have “worse dying experiences” [84]. Patients with advanced COPD are more likely to undergo invasive procedures such as mechanical ventilation and are less likely to receive hospice care and end-of-life planning compared to patients with lung cancer [85]. Thus, delaying access to end-of-life care reduces the ability to increase the quality of life and promote end-of-life discussions [83].

Clinicians play a central role in the quality of care received during the end stages of COPD, but there are major barriers that they face toward providing quality end-of-life care for patients with advanced COPD. These barriers include a lack of continuity of care affecting the relationship of physicians and patients, as well as a lack of education on the roles and goals of palliative care. Most patients would like to receive comprehensive and accurate information about disease progression, treatment options, and prognosis; however, this information is often lacking [73]. Pulmonologists have skills and expertise for overcoming difficulties in COPD diagnosis and for providing the best treatment for managing COPD. Many pulmonology providers have acknowledged misconceptions toward palliative care, including the fear that palliative care clinicians would manage symptoms with high doses of opioids and benzodiazepines. Other barriers include a lack of education regarding when and how to have end-of-life discussions with patients [83]. Poor communication is more likely to lead to poor palliative care in COPD [8]. Despite these barriers, it has been shown that COPD patients benefit from prompt access to palliative care [74]. The goal of palliative care is to prevent and relieve suffering from COPD exacerbation regardless of the stage of the disease [86]. However, the unpredictable nature of disease progression in patients with COPD makes proper planning of end-of-life care difficult. This unpredictability is further complicated during periods of acute exacerbations, where an initial complaint of respiratory discomfort can progress rapidly into respiratory failure. This often puts the patient in an incapacitated state, depriving them of the opportunity to actively participate in the process of end-of-life care planning. For this reason, improving prognostication skills is key, so clinicians may identify COPD patients that are at greater risk for worse outcomes, and promptly commence end-of-life care planning from the initial encounter [87].

**Figure 1:**
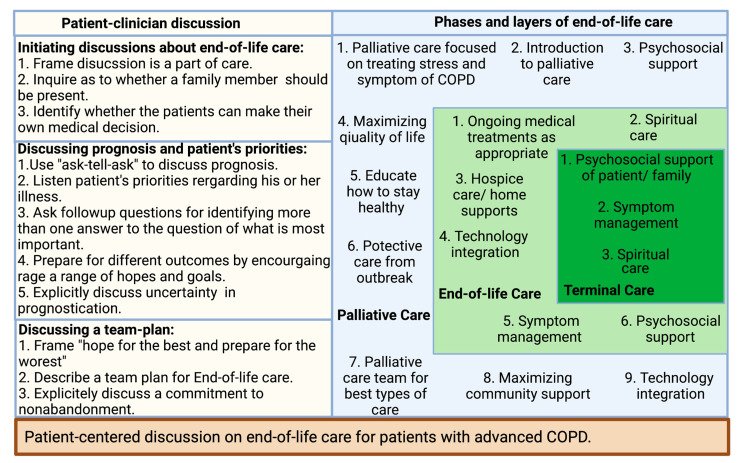
Patient-centered discussion on end-of-life care for patients with advanced COPD.

Many clinicians find communicating about end-of-life care difficult due to the nature of the discussion, especially with patients in the early stages of COPD. Therefore, adopting the right communication technique is crucial in the overall improvement of end-of-life care (Figure 1) [8]. Patients with COPD more than often want to know information about five specific areas: diagnosis and disease process, treatment, prognosis, what dying might be like, and advance care planning [8]. There are several published studies that provide some specific directions for clinicians in their communications about end-of-life discussion to their patients with advanced COPD. For example, most COPD patients would like more information about prognosis, while patients with advanced COPD and limited life expectancy want to know more about their life expectancy [88,89,90]. It is not unusual that family members request this information even the patient themself does not want to know [91]. Therefore, clinicians should be aware of patients’ and family members’ interests in obtaining information about the disease. It has been found that describing information about a disease with numeric experiences of risk (e.g., eight out of ten people will have side effects from this drug) allowed for better comprehension than describing information about a disease with qualitative expressions of risk [92].

Patients with COPD may receive poor-quality palliative care due to the lack of patient–physician communication about end-of-life care, or if it occurs too late in the illness [93,94,95]. By understanding the barriers to communication, clinicians can improve patient–clinician discussions about end-of-life care [8]. A recent study suggests that inpatient palliative care consultation has a positive impact on patient outcomes and transitions to the community [96]. A short survey from both pulmonary and palliative clinicians suggested early care adds value to disease-focused COPD care. In the study, the clinicians from both specialties not only supported early palliative care in COPD but also emphasized addressing pulmonologists’ misconception of palliative care, establishing consensus referral criteria, and executing a novel early palliative care model [83]. Clinicians should be prepared to listen and provide appropriate information in the most caring and respectful manner. Practical strategies that can be employed begin with building a relationship based on trust between a COPD patient and their provider. Discussions about end-of-life care must be initiated during the early course of the disease focusing on the implication of the patient’s diagnosis and interventions to be taken at every step of disease progression [97]. A phased introduction of supportive and palliative care can be triggered at key disease milestones during a lifelong journey with COPD, especially during an inpatient visit for an exacerbation [98]. To ensure patients’ wishes regarding end-of-life care are current, the advanced care directive should be reviewed at every visit [97]. Clinician–patient conversations can bring a successful end-of-life care plan with active participation by patients, clinicians, and caregivers (Figure 1) [8].

## 7. Prospective

COPD affects the lives of many, but with proper diagnosis, treatment, and planning of care, COPD patients can lead relatively normal lives. It is vital that clinicians utilize spirometry as well as physical exam findings to make a diagnosis of COPD. Clinicians can prescribe a broad range of medications to ease and prevent symptoms and exacerbations of advanced COPD. Clinicians should make every effort to initiate end-of-life care planning at an appropriate time so that the patient’s requests regarding pain management and medical interventions can be honored. By taking a holistic approach to understanding the prevalence, pathophysiology, treatment options, and end-of-life considerations for COPD, clinicians can effectively manage severe cases of COPD and maintain trusting professional relationships with their patients.

## References

[1] GOLD 2021 Global Strategy for Prevention, Diagnosis and Management of COPD; In 2021 GOLD Report. 2021, Global Initiative for Chronic Obstructive Lung Disease—GOLD. https://goldcopd.org/wp-content/uploads/2020/11/GOLD-REPORT-2021-v1.1-25Nov20_WMV.pdf.

[2] Silverman E.K., Crapo J.D., Make B.J., Jameson J., Fauci A.S., Kasper D.L., Hauser S.L., Longo D.L., Loscalzo J. (2018). Chronic obstructive pulmonary disease. Harrison’s Principles of Internal Medicine.

[3] Barnes P.J. (2016). Sex Differences in Chronic Obstructive Pulmonary Disease Mechanisms. Am. J. Respir. Crit. Care. Med..

[4] Sin D.D., Anthonisen N.R., Soriano J.B., Agusti A.G. (2006). Mortality in COPD: Role of comorbidities. Eur. Respir. J..

[5] Iglesias J.R., Díez-Manglano J., García F.L., Peromingo J.A.D., Almagro P., Aguilar J.M.V. (2020). Management of the COPD Patient with Comorbidities: An Experts Recommendation Document. Int. J. Chron. Obs. Pulmon. Dis..

[6] Díez-Manglano J., López-García F. (2014). Protocolos: Manejo Diagnóstico y Terapéutico de las Comorbilidades en la EPOC [Protocols: Diagnostic and Therapeutic management of Comorbidities in COPD].

[7] Zhou H.-X., Ou X.-M., Tang J.-Y., Wang L., Feng Y.-L. (2015). Advanced Chronic Obstructive Pulmonary Disease: Innovative and Integrated Management Approaches. Chin. Med. J..

[8] Curtis J.R. (2008). Palliative and end-of-life care for patients with severe COPD. Eur. Respir. J..

[9] Weingaertner V., Scheve C., Gerdes V., Schwarz-Eywill M., Prenzel R., Bausewein C., Higginson I.J., Voltz R., Herich L., Simon S.T. (2014). Breathlessness, functional status, distress, and palliative care needs over time in patients with advanced chronic obstructive pulmonary disease or lung cancer: A cohort study. J. Pain Symptom Manag..

[10] Janssen D.J., Spruit M.A., Uszko-Lencer N.H., Schols J.M., Wouters E.F. (2011). Symptoms, Comorbidities, and Health Care in Advanced Chronic Obstructive Pulmonary Disease or Chronic Heart Failure. J. Palliat. Med..

[11] Okutan O., Tas T., Demirer E., Kartalogu Z. (2013). Evaluation of quality of life with the chronic obstructive pulmonary disease assessment test in chronic obstructive pulmonary disease and the effect of dyspnea on disease-specific quality of life in these patients. Yonsei Med. J..

[12] Pellegrino R., Viegi R., Brusasco V., Crapo R.O., Burgos F., Casaburi R., Coates A., van der Grinten C.P.M., Gustafsson P., Jensen R. (2005). Interpretative strategies for lung function tests. Eur. Respir. J..

[13] Anzueto A., Miravitlles M. (2017). Pathophysiology of dyspnea in COPD. Postgrad Med..

[14] Gundry S. (2019). COPD 1: Pathophysiology, diagnosis and prognosis. Nurs. Times.

[15] Kent B.D., Mitchell P.D., McNicholas W.T. (2011). Hypoxemia in patients with COPD: Cause, effects, and disease progression. Int. J. Chron. Obs. Pulmon. Dis..

[16] Barnes P.J., Shapiro S.D., Pauwels R.A. (2003). Chronic obstructive pulmonary disease: Molecular and cellular mechanisms. Eur. Respir. J..

[17] Senior R.M., Pierce R.A., Atkinson J.J., Grippi M.A., Elias J.A., Fishman J.A., Kotloff R.M., Pack A.I., Senior R.M., Siegel M.D. (2015). Chronic obstructive pulmonary disease: Epidemiology, pathophysiology, pathogenesis, and α1-antitrypsin deficiency. Fishman’s Pulmonary Diseases and Disorders.

[18] Lareau S., Moseson E., Slatore C.G. (2018). Exacerbation of COPD. Am. J. Respir. Crit. Care Med..

[19] Bhat T.A., Panzica L., Kalathil S.G., Thanavala Y. (2015). Immune Dysfunction in Patients with Chronic Obstructive Pulmonary Disease. Ann. Am. Thorac. Soc..

[20] Niewoehner D.E. (2006). The Impact of Severe Exacerbations on Quality of Life and the Clinical Course of Chronic Obstructive Pulmonary Disease. Am. J. Med..

[21] Raluy-Callado M., Lambrelli D., MacLachlan S., Khalid J.M. (2015). Epidemiology, severity, and treatment of chronic obstructive pulmonary disease in the United Kingdom by GOLD 2013. Int. J. Chronic Obstr. Pulm. Dis..

[22] Ball P. (1995). Epidemiology and Treatment of Chronic Bronchitis and Its Exacerbations. Chest.

[23] Sunyer J., Sáez M., Murillo C., Castellsague J., Martínez F., Antó J.M. (1993). Air pollution and emergency room admissions for chronic obstructive pulmonary disease: A 5-year study. Am. J. Epidemiol..

[24] Connors A.F., Dawson N.V., Thomas C., Harrell F.E., Desbiens N., Fulkerson W.J., Kussin P., Bellamy P., Goldman L., Knaus W.A. (1996). Outcomes following acute exacerbation of severe chronic obstructive lung disease. The SUPPORT investigators (Study to Understand Prognoses and Preferences for Outcomes and Risks of Treatments). Am. J. Respir. Crit. Care Med..

[25] Ritchie A.I., Wedzicha J.A. (2020). Definition, Causes, Pathogenesis, and Consequences of Chronic Obstructive Pulmonary Disease Exacerbations. Clin. Chest Med..

[26] Alobaidi N.Y., James A.S., Stockley A.R., Sapey E. (2020). An overview of exacerbations of chronic obstructive pulmonary disease: Can tests of small airways’ function guide diagnosis and management?. Ann. Thorac. Med..

[27] Sapey E., Stockley R.A. (2006). COPD exacerbations. 2: Aetiology. Thorax.

[28] Jafarinejad H., Moghoofei M., Mostafaei S., Salimian J., Jamalkandi S.A., Ahmadi A. (2017). Worldwide prevalence of viral infection in AECOPD patients: A meta-analysis. Microb. Pathog..

[29] Mohan A., Chandra S., Agarwal D., Guleria R., Broor S., Gaur B., Pandey R.M. (2010). Prevalence of viral infection detected by PCR and RT-PCR in patients with acute exacerbation of COPD: A systematic review. Respirology.

[30] Choi M.E., Price D.R., Ryter S.W., Choi A.M.K. (2019). Necroptosis: A crucial pathogenic mediator of human disease. JCI Insight.

[31] D’Anna S.E., Maniscalco M., Cappello F., Carone M., Motta A., Balbi B., Ricciardolo F.L.M., Caramori G., Di Stefano A. (2020). Bacterial and viral infections and related inflammatory responses in chronic obstructive pulmonary disease. Ann. Med..

[32] Lee S., Hirohama M., Noguchi M., Nagata K., Kawaguchi A. (2018). Influenza A Virus Infection Triggers Pyroptosis and Apoptosis of Respiratory Epithelial Cells through the Type I Interferon Signaling Pathway in a Mutually Exclusive Manner. J. Virol..

[33] Groskreutz D.J., Monick M.M., Yarovinsky T.O., Powers L.S., Quelle D., Varga S., Look D.C., Hunninghake G.W. (2007). Respiratory Syncytial Virus Decreases p53 Protein to Prolong Survival of Airway Epithelial Cells. J. Immunol..

[34] Li M., Li J., Zeng R., Yang J., Liu J., Zhang Z., Song X., Yao Z., Ma C., Li W. (2018). Respiratory Syncytial Virus Replication Is Promoted by Autophagy-Mediated Inhibition of Apoptosis. J. Virol..

[35] da Silva M.C., Zahm J.M., Gras D., Bajolet O., Abely M., Hinnrasky J., Milliot M., de Assis M.C., Hologne C., Bonnet N. (2004). Dynamic interaction between airway epithelial cells and Staphylococcus aureus. Am. J. Physiol. Lung Cell Mol. Physiol..

[36] DD’Anna S.E., Balbi B., Cappello F., Carone M., Di Stefano A. (2016). Bacterial–viral load and the immune response in stable and exacerbated COPD: Significance and therapeutic prospects. Int. J. Chronic Obstr. Pulm. Dis..

[37] Leigh R., Proud D. (2014). Virus-induced modulation of lower airway diseases: Pathogenesis and pharmacologic approaches to treatment. Pharmacol. Ther..

[38] Wilkinson T.M., Hurst J.R., Perera W.R., Wilks M., Donaldson G.C., Wedzicha J.A. (2006). Effect of Interactions Between Lower Airway Bacterial and Rhinoviral Infection in Exacerbations of COPD. Chest.

[39] Kiser T., Allen R.R., Valuck R.J., Moss M., Vandivier R.W. (2014). Outcomes Associated with Corticosteroid Dosage in Critically Ill Patients with Acute Exacerbations of Chronic Obstructive Pulmonary Disease. Am. J. Respir. Crit. Care Med..

[40] Su J., Liu H.-Y., Tan X.-L., Ji Y., Jiang Y.-X., Prabhakar M., Rong Z.-H., Zhou H.-W., Zhang G.-X. (2015). Sputum Bacterial and Fungal Dynamics during Exacerbations of Severe COPD. PLoS ONE.

[41] Pashley C.H. (2014). Fungal culture and sensitisation in asthma, cystic fibrosis and chronic obstructive pulmonary disorder: What does it tell us?. Mycopathologia.

[42] Patel D.A., Gao X., Stephens J.M., Forshag M.S., Tarallo M. (2011). US hospital database analysis of invasive aspergillosis in the chronic obstructive pulmonary disease non-traditional host. J. Med. Econ..

[43] CDC (2020). People with Certain Medical Conditions. https://www.cdc.gov/coronavirus/2019-ncov/need-extra-precautions/people-with-medical-conditions.html.

[44] Sin D.D. (2020). COVID-19 in COPD: A growing concern. eClinicalMedicine.

[45] Leung J.M., Niikura M., Yang C.W.T., Sin D.D. (2020). COVID-19 and COPD. Eur. Respir. J..

[46] Osan J.K., Talukdar S.N., Feldmann F., DeMontigny B.A., Jerome K., Bailey K.L., Feldmann H., Mehedi M. (2020). Goblet Cell Hyperplasia Increases SARS-CoV-2 Infection in COPD. bioRxiv.

[47] Mehedi M. (2020). Goblet Cells in SARS-CoV-2 Pathogenesis. AJBSR.

[48] Martines R.B., Ritter J.M., Matkovic E., Gary J., Bollweg B.C., Bullock H., Goldsmith C.S., Silva-Flannery L., Seixas J.N., Reagan-Steiner S. (2020). Pathology and Pathogenesis of SARS-CoV-2 Associated with Fatal Coronavirus Disease, United States. Emerg. Infect. Dis..

[49] Decramer M., Janssens W., Miravitlles M. (2012). Chronic obstructive pulmonary disease. Lancet.

[50] Riley C.M., Sciurba F.C. (2019). Diagnosis and Outpatient Management of Chronic Obstructive Pulmonary Disease: A Review. JAMA.

[51] Halpin D.M., Miravitlles M., Metzdorf N., Celli B. (2017). Impact and prevention of severe exacerbations of COPD: A review of the evidence. Int. J. Chron. Obs. Pulmon. Dis..

[52] Hsu E., Bajaj T. (2021). Beta 2 Agonists.

[53] Salpeter S.R., Ormiston T.M., Salpeter E.E. (2004). Cardiovascular effects of beta-agonists in patients with asthma and COPD: A meta-analysis. Chest.

[54] Bhakta N.R., Trevor A.J., Katzung B.G., Kruidering-Hall M.M., Masters S.B. (2021). Drugs used in asthma & chronic obstructive pulmonary disease. Basic & Clinical Pharmacology.

[55] Scullion J.E. (2007). The development of anticholinergics in the management of COPD. Int. J. Chron. Obs. Pulmon. Dis..

[56] Antus B. (2013). Pharmacotherapy of Chronic Obstructive Pulmonary Disease: A Clinical Review. Int. Sch. Res. Not..

[57] Alagha K., Palot A., Sofalvi T., Pahus L., Gouitaa M., Tummino C., Martinez S., Charpin D., Bourdin A., Chanez P. (2014). Long-acting muscarinic receptor antagonists for the treatment of chronic airway diseases. Ther. Adv. Chronic Dis..

[58] Suissa S., McGhan R., Niewoehner D., Make B. (2007). Inhaled corticosteroids in chronic obstructive pulmonary disease. Proc. Am. Thorac. Soc..

[59] Jarad N.A., Wedzicha J.A., Burge P.S., Calverley P.M. (1999). An observational study of inhaled corticosteroid withdrawal in stable chronic obstructive pulmonary disease. ISOLDE Study Group. Respir. Med..

[60] Tashkin D.P., Strange C. (2018). Inhaled corticosteroids for chronic obstructive pulmonary disease: What is their role in therapy?. Int. J. Chron. Obs. Pulmon. Dis..

[61] Gottwalt B., Tadi P. (2021). Methylxanthines.

[62] Vanfleteren L., Fabbri L.M., Papi A., Petruzzelli S., Celli B. (2018). Triple therapy (ICS/LABA/LAMA) in COPD: Time for a reappraisal. Int. J. Chron. Obs. Pulmon. Dis..

[63] Zheng Y., Zhu J., Liu Y., Lai W., Lin C., Qiu K., Wu J.J., Yao W. (2018). Triple therapy in the management of chronic obstructive pulmonary disease: Systematic review and meta-analysis. BMJ.

[64] Martinez F.J., Rabe K.F., Ferguson G.T., Wedzicha J.A., Trivedi R., Jenkins M., Darken P., Aurivillius M., Dorinsky P. (2021). Benefits of budesonide/glycopyrrolate/formoterol fumarate (BGF) on symptoms and quality of life in patients with COPD in the ETHOS trial. Respir. Med..

[65] Vanfleteren L., Ullman A., Nordenson A., Andersson A., Andelid K., Fabbri L.M. (2019). Triple therapy (ICS/LABA/LAMA) in COPD: Thinking out of the box. ERJ Open Res..

[66] Ahmed S.M., Athar M. (2015). Mechanical ventilation in patients with chronic obstructive pulmonary disease and bronchial asthma. Indian J. Anaesth..

[67] Carlin B.W., Wiles K.S., McCoy R.W., Brennan T., Easley D., Thomashow R. (2015). Effects of a Highly Portable Noninvasive Open Ventilation System on Activities of Daily Living in Patients with COPD. Chronic Obs. Pulm. Dis..

[68] Berggren J. Home ventilators keep patients healthy and out of the hospital. Breathe Easy. Home Medical Services.

[69] Lacasse Y., Tan A.M., Maltais F., Krishnan J.A. (2018). Home Oxygen in Chronic Obstructive Pulmonary Disease. Am. J. Respir. Crit. Care Med..

[70] Cranston J.M., Crockett A.J., Moss J.R., Alpers J.H. (2005). Domiciliary oxygen for chronic obstructive pulmonary disease. Cochrane Database Syst. Rev..

[71] Franssen F.M., Alter P., Bar N., Benedikter B.J., Iurato S., Maier D., Maxheim M., Roessler F.K., Spruit M.A., Vogelmeier C.F. (2019). Personalized medicine for patients with COPD: Where are we?. Int. J. Chron. Obs. Pulmon. Dis..

[72] Ambrosino N., Bertella E. (2018). Lifestyle interventions in prevention and comprehensive management of COPD. Breathe.

[73] Tavares N., Hunt K.J., Jarrett N., Wilkinson T.M. (2020). The preferences of patients with chronic obstructive pulmonary disease are to discuss palliative care plans with familiar respiratory clinicians, but to delay conversations until their condition deteriorates: A study guided by interpretative phenomenological analysis. Palliat. Med..

[74] Smallwood N., Currow D., Booth S., Spathis A., Irving L., Philip J. (2018). Attitudes to specialist palliative care and advance care planning in people with COPD: A multi-national survey of palliative and respiratory medicine specialists. BMC Palliat. Care.

[75] Kuzma A.M., Meli Y., Meldrum C., Jellen P., Butler-Lebair M., Koczen-Doyle D., Rising P., Stavrolakes K., Brogan. F. (2008). Multidisciplinary care of the patient with chronic obstructive pulmonary disease. Proc. Am. Thorac. Soc..

[76] Team of Specialists. https://www.nationaljewish.org/directory/copd/team.

[77] Family Members Share in the Care of COPD Patients. https://www.medicaleconomics.com/view/family-members-share-care-copd-patients..

[78] Tonelli M.R. (1996). Pulling the plug on living wills. A critical analysis of advance directives. Chest.

[79] Murray S.A., Kendall M., Boyd K., Sheikh A. (2005). Illness trajectories and palliative care. BMJ.

[80] Dales R.E., O’Connor A., Hebert P., Sullivan K., Mc Kim D., Llewellyn-Thomas H. (1999). Intubation and mechanical ventilation for COPD: Development of an instrument to elicit patient preferences. Chest.

[81] Tilden V.P., Tolle S.W., Drach L.L., Perrin N.A. (2004). Out-of-hospital death: Advance care planning, decedent symptoms, and caregiver burden. J. Am. Geriatr. Soc..

[82] Norris K., Merriman M.P., Curtis J.R., Asp C., Tuholske L., Byock I.R. (2007). Next of kin perspectives on the experience of end-of-life care in a community setting. J. Palliat. Med..

[83] Iyer A.S., Dionne-Odom J.N., Khateeb D.M., O’Hare L., Tucker R.O., Brown C.J., Dransfield M.T., Bakitas M.A. (2020). A Qualitative Study of Pulmonary and Palliative Care Clinician Perspectives on Early Palliative Care in Chronic Obstructive Pulmonary Disease. J. Palliat. Med..

[84] Kendzerska T., Nickerson J.W., Hsu A.T., Gershon A.S., Talarico R., Mulpuru S., Pakhale S., Tanuseputro P. (2019). End-of-life care in individuals with respiratory diseases: A population study comparing the dying experience between those with chronic obstructive pulmonary disease and lung cancer. Int. J. Chron. Obs. Pulmon. Dis..

[85] Fu P.K., Yang M.C., Wang C.Y., Lin S.P., Kuo C.T., Hsu C.Y., Tung Y.C. (2019). Early Do-Not-Resuscitate Directives Decrease Invasive Procedures and Health Care Expenses During the Final Hospitalization of Life of COPD Patients. J. Pain Symptom Manag..

[86] American Academy of Hospice and Palliative Medicine, Center to Advance Palliative Care, Hospice and Palliative Nurses Association, Last Acts Partnership, National Hospice and Palliative Care Organization (2004). National consensus project for quality palliative care: Clinical practice guidelines for quality palliative care, executive summary. J. Palliat. Med..

[87] Spathis A., Booth S. (2008). End of life care in chronic obstructive pulmonary disease: In search of a good death. Int. J. Chron. Obs. Pulmon. Dis..

[88] Curtis J.R., Wenrich M.D., Carline J.D., Shannon S.E., Ambrozy D.M., Ramsey P.G. (2002). Patients’ perspectives on physician skill in end-of-life care: Differences between patients with COPD, cancer, and AIDS. Chest.

[89] Jones I., Kirby A., Ormiston P., Loomba Y., Chan K.K., Rout J., Nagle J., Wardman L., Hamilton S. (2004). The needs of patients dying of chronic obstructive pulmonary disease in the community. Fam. Pr..

[90] Fried T.R., Bradley E.H., O’Leary J. (2003). Prognosis communication in serious illness: Perceptions of older patients, caregivers, and clinicians. J. Am. Geriatr. Soc..

[91] Curtis J.R., Engelberg R., Young J.P., Vig L.K., Reinke L.F., Wenrich M.D., McGrath B., McCown E., Back A.L. (2008). An approach to understanding the interaction of hope and desire for explicit prognostic information among individuals with severe chronic obstructive pulmonary disease or advanced cancer. J. Palliat. Med..

[92] Paling J. (2003). Strategies to help patients understand risks. BMJ.

[93] Curtis J., Engelberg R., Nielsen E., Au D., Patrick D. (2004). Patient-physician communication about end-of-life care for patients with severe COPD. Eur. Respir. J..

[94] Heffner J.E., Fahy B., Hilling L., Barbieri C. (1997). Outcomes of advance directive education of pulmonary rehabilitation patients. Am. J. Respir. Crit. Care Med..

[95] Heffner J.E., Fahy B., Hilling L., Barbieri C. (1996). Attitudes regarding advance directives among patients in pulmonary rehabilitation. Am. J. Respir. Crit. Care Med..

[96] Scott M., Shaver N., Lapenskie J., Isenberg S.R., Saunders S., Hsu A.T., Tanuseputro P. (2020). Does inpatient palliative care consultation impact outcomes following hospital discharge? A narrative systematic review. Palliat. Med..

[97] Halpin D.M.G., Dionne-Odom J.N., Khateeb D.M., O’Hare L., Tucker R.O., Brown C.J., Dransfield M.T., Bakitas M.A. (2018). Palliative care for people with COPD: Effective but underused. Eur. Respir. J..

[98] Pinnock H., Kendall M., Murray S.A., Worth A., Levack P., Porter M., MacNee W., Sheikh A. (2011). Living and dying with severe chronic obstructive pulmonary disease: Multi-perspective longitudinal qualitative study. BMJ.

